# DIGITAL DETECTION OF DEMENTIA (D CUBED) TRIALS

**DOI:** 10.1093/geroni/igae098.1928

**Published:** 2024-12-31

**Authors:** Malaz Boustani, Nicole Fowler

**Affiliations:** Indiana University Indianapolis, Indianapolis, Indiana, United States; Indiana University, Indianapolis, Indiana, United States

## Abstract

At Indiana University, we leveraged electronic health record (EHR) data and Machine Learning algorithms to develop a Passive Digital Marker (PDM) for dementia detection with 80% accuracy. At the University of Miami, we developed the Quick Dementia Rating Scale (QDRS) as a 2-3 minute patient-reported outcome tool with 85% accuracy for dementia diagnosis. Two pragmatic cluster-randomized controlled comparative effectiveness trials randomized primary care clinics to Usual care, PDM alone, and both the PDM and the QDRS. Trials recruited diverse rural, suburban and urban primary care practices located in central Indiana and south Florida. The positive results are communicated via the EHR only to clinicians within the PDM and PDM + QDRS clinics during an ambulatory primary care visit. The primary outcome measure is the annual rate of new documented dementia diagnosis. In the first year, the Indiana trial enrolled 5960 older patients with a mean age of 71.9 years and SD of 6.50, 61% were female and 51% were African American. In the clinics that were randomized to PDM + QDRS, there were 827 patients who completed at least one question on the QDRS. The Indiana trial used a non-interruptive Best Practice Alert (BPA) for the first year of operation. For the second year of operation, the trial coupled the BPA with an InBasket message to primary care physicians to overcome the shortcoming of the Non-interruptive nature of the BPA. We have created an IT implementation manual to deploy the PDM with minimum expertise.

